# The Legacy of Innovation: A Comprehensive Review of Eponymous Procedures in Thoracic, Congenital, and Pediatric Heart Diseases

**DOI:** 10.7759/cureus.83831

**Published:** 2025-05-10

**Authors:** Sachin Talwar, Krishnan Ganapathy Subramaniam, Vishal V Bhende, Mathangi Krishnakumar

**Affiliations:** 1 Cardiothoracic and Vascular Surgery, All India Institute of Medical Sciences, New Delhi, IND; 2 Pediatric Cardiac Surgery, Sri Padmavati Pediatric Heart Centre, Sri Venkateswara Institute of Medical Sciences (SVIMS) Campus, Tirupati, IND; 3 Pediatric Cardiac Surgery, Bhanubhai and Madhuben Patel Cardiac Centre, Shree Krishna Hospital, Bhaikaka University, Karamsad, IND; 4 Anaesthesiology, St John's Medical College Hospital, Bengaluru, IND

**Keywords:** bhende-pathak hernia, blalock-taussig, damus-kaye-stansel(dks), fontan, jatene, kawashima, lecompte, mustard, norwood, potts

## Abstract

Eponymous cardiovascular surgeries have played an important role in the evolution of congenital and pediatric heart disease management. These procedures, named after pioneering surgeons, have significantly advanced surgical interventions for complex cardiac conditions. This review provides an overview of eponymous cardiovascular surgeries and their impact on managing congenital and pediatric heart diseases. We examine these procedures’ historical significance, technical advancements, and evolving role in contemporary surgical practice. Historically, operations such as the Blalock-Taussig-Thomas shunt and the Fontan procedure have provided life-saving solutions for patients with congenital heart defects. Over time, many eponymous procedures have been refined or replaced as surgical techniques and technologies have evolved. For example, the Jatene procedure has largely supplanted the Mustard and Senning operations to transpose the great arteries, offering superior long-term outcomes. Similarly, in conditions of functional or anatomical absence of one ventricle, iterative improvements in the Fontan and Glenn procedures have enhanced survival rates and reduced complications. The advent of catheter-based interventions, hybrid surgical techniques, and three-dimensional imaging has further transformed the field, improving surgical precision and patient outcomes. Despite modifications and replacements, the legacy of these eponymous procedures remains foundational to cardiovascular surgery. Continued innovation, collaboration, and research will build upon these historical milestones, ensuring ongoing advancements in treating congenital and pediatric heart diseases.

## Introduction and background

Eponyms in medicine honor pioneering individuals who have shaped modern medical and surgical practices. By incorporating their names into diseases, techniques, and procedures, eponyms provide historical context and highlight the lasting impact of their contributions. In cardiovascular surgery, particularly in treating congenital and pediatric heart diseases, eponymous procedures have transformed the management of conditions once considered inoperable or fatal. These procedures remain essential to modern cardiothoracic surgery, improving survival and quality of life.

Congenital heart disorders are among the most common congenital malformations, affecting approximately one in 100 live births worldwide. Advances in neonatal and pediatric cardiology and innovative surgical techniques have significantly improved survival rates, allowing many patients to reach adulthood [1,2]. Historically, individuals with cyanotic and acyanotic heart defects had limited treatment options and often did not survive infancy or early childhood. This review examines eponymous cardiovascular surgeries’ origins, technical innovations, and clinical impact. It highlights the ingenuity of the surgeons who developed these procedures and explores their enduring influence on congenital and pediatric cardiology. From the Blalock-Taussig shunt, which revolutionized the prognosis of tetralogy of Fallot (TOF), to the Fontan procedure, which provided a treatment option for single-ventricle physiology, each eponym represents a significant milestone in medical history.

## Review

Literature search and selection criteria

A narrative literature review was conducted to identify and describe historically significant eponymous surgical procedures. Electronic searches were performed in PubMed and Scopus databases up to January 2025, using combinations of keywords including “eponymous procedure”, “Blalock-Taussig”, “Fontan”, “Glenn”, “historic surgery”, and “surgical technique”. Inclusion criteria encompassed peer-reviewed articles describing the origin, technique, clinical application, or evolution of eponymous surgical interventions. Articles not published in English, abstracts without full-text availability, and those not directly addressing cardiovascular procedures were excluded. Selected studies were reviewed for historical context, procedural description, and current relevance to contemporary surgical practice, aiming to ensure a representative and informative overview.

Pioneering palliative shunts

Blalock-Taussig Shunt

Introduced in 1944, the Blalock-Taussig shunt marked the advent of modern pediatric cardiac surgery. Alfred Blalock, in collaboration with Helen Taussig and Vivien Thomas, developed a shunt to permit systemic to pulmonary blood flow among patients with cyanotic heart disease, particularly TOF. The procedure involved connecting the subclavian artery and the pulmonary artery (PA), mimicking a patent ductus arteriosus [3]. This innovation provided the first effective palliation for “blue baby syndrome,” significantly improving survival and establishing a foundation for future advancements in congenital heart surgery [4].

Pott’s Shunt 

Developed by Willis Potts in 1946, the Pott’s shunt served as an alternative to the Blalock-Taussig shunt for patients in whom the latter was technically unfeasible. This anastomosis across the left PA and the descending aorta, made between the sides of the two vessels, provided additional blood flow from the systemic to the pulmonary circulation in conditions of pulmonary stenosis and tricuspid atresia (TA) among neonates [5]. Its lower risk of thrombosis and avoidance of small-caliber vessels made it a valuable option for specific cases [5].

Waterston Shunt

In 1962, David Waterston introduced the Waterston shunt, an anastomosis between the side of the ascending aorta and the side of the right PA. Designed as a palliative procedure for the TOF, it increased pulmonary blood flow while avoiding complications associated with subclavian artery utilization in the Blalock-Taussig shunt. The Waterston shunt was particularly beneficial for patients with complex congenital defects [6].

Innovations in staged palliation

Glenn Procedure

William Glenn introduced the Glenn procedure in 1958 as a groundbreaking cavopulmonary anastomosis, a technique that bypassed the right atrium by connecting the superior vena cava to the right PA, to establish a low-pressure pulmonary circulation [7]. The original Glenn shunt evolved into the bidirectional Glenn shunt (BDG), which is now a critical intermediate stage in the palliation of single-ventricle (SV) physiology [8].

Fontan Procedure

Described by Francis Fontan in 1971, the Fontan procedure represents a landmark innovation in the management of SV physiology. The procedure reroutes systemic venous return from the inferior vena cava directly to the pulmonary circulation, bypassing the right heart, addressing the hemodynamic challenges of TA and hypoplastic left heart syndrome (HLHS) [9]. Over time, the Fontan procedure has undergone significant modifications, including staged approaches incorporating the BDG as an intermediary step, improving outcomes and reducing complications [10].

Kawashima Procedure

In 1984, Yasunaru Kawashima developed the Kawashima procedure by modifying the Fontan procedure to suit interrupted inferior vena cava and azygos continuation conditions. The Kawashima procedure involves a bilateral bidirectional Glenn shunt to manage single ventricle pathologies with bilateral SVCs (right and left SVC) and complex SV physiologies, particularly in polysplenia syndrome [11]. This approach optimizes Fontan circulation by addressing specific anatomical challenges and improving long-term outcomes [12].

Norwood Procedure

Introduced by William Norwood in 1983, the Norwood procedure revolutionized the management of HLHS. This staged approach reconstructs systemic circulation by creating a neo-aorta and constructs a Blalock-Taussig shunt or a Sano shunt for pulmonary circulation. The Norwood procedure remains the cornerstone of HLHS palliation, significantly improving survival rates for this previously fatal condition [13].

Sano Shunt

Shunji Sano’s modification of the Norwood procedure, introduced in 2003, replaced the traditional Blalock-Taussig shunt (which connects the subclavian artery to the PA) by connecting the right ventricle to the PA. The Sano shunt enhances pulmonary blood flow and reduces the risk of the coronary steal phenomenon, improving outcomes in the first stage of HLHS palliation [14].

Definitive repairs

Damus-Kaye-Stansel (DKS) Procedure

In the 1970s, Paul Damus, Michael Kaye, and Horace Stansel described a procedure to facilitate systemic circulation where there is a ventricular septal defect (VSD) with transposition of the great arteries (TGA). By anastomosing the PA to the ascending aorta, the DKS procedure ensures unobstructed systemic blood flow while allowing for subsequent palliation or definitive repair [15].

Jatene Procedure

Adib Jatene introduced the arterial switch operation in 1975 to replace atrial switch techniques such as the Mustard and Senning procedures developed to address dextro-transposition of the great arteries (D-TGA) by including an additional procedure to reimplant the coronary arteries. This anatomically corrective procedure involves transecting and repositioning the great arteries while reimplanting the coronary arteries [16]. The Jatene procedure, often performed with the Lecompte maneuver, remains the standard of care for D-TGA correction [17].

Rastelli Procedure

Giancarlo Rastelli developed the Rastelli procedure in 1969 to treat VSD with left ventricular outflow tract obstruction and D-TGA. This technique uses a synthetic or homograft conduit to direct blood from the left ventricle through the VSD into the aorta while routing pulmonary outflow through an extracardiac conduit to the PAs [18]. The Rastelli procedure provided a definitive repair for patients previously considered inoperable.

Ross Procedure

Introduced by Donald Ross in 1967 to address valvular heart disease of the aortic valves in pediatric patients and young adults, this procedure uses an autograft of the pulmonary valves to replace the diseased aortic valve and replaces the patient’s pulmonary valves with a pulmonary allograft. The Ross procedure provides excellent hemodynamic outcomes and durability, particularly in pediatric patients, where growth potential is critical [19].

Specialized interventions

Mustard and Senning Procedures

These atrial switch procedures were developed in the mid-20th century for D-TGA. The Mustard procedure, introduced by William Mustard in 1964, used synthetic materials to redirect systemic and pulmonary venous returns to their appropriate ventricles [20]. The Senning procedure, first performed by Åke Senning in 1957, relied on native atrial tissue for a similar redirection [21]. While effective, both procedures were associated with long-term complications, including arrhythmias and right ventricular dysfunction [22].

Bentall Procedure

In 1968, Hugh Bentall and Antony De Bono introduced the Bentall procedure to address aortic root aneurysms or combined aortic valve disease and ascending aortic pathology. The Bentall procedure uses a composite graft to replace the diseased aortic root and valve while reimplanting the coronary arteries. The Bentall procedure remains a standard technique for managing complex aortic root disease, with modifications improving safety and efficacy [23].

Lecompte Maneuver

Yves Lecompte introduced the Lecompte maneuver in 1981 as a critical component of the arterial switch operation for D-TGA. This technique involves repositioning the PAs anterior to the aorta, reducing tension on the coronary arteries, and improving anatomic alignment. The Lecompte maneuver is now a standard step in the Jatene procedure, enhancing long-term outcomes in neonates undergoing arterial switch surgery [24].

Rashkind Procedure

William Rashkind developed the Rashkind balloon atrial septostomy in 1966 as a palliative intervention for neonates with TGA or other cyanotic heart defects. This catheter-based procedure creates or enlarges an atrial septal defect to improve oxygenation by allowing mixing of oxygenated and deoxygenated blood. The Rashkind procedure was pivotal in interventional cardiology, offering a minimally invasive option for critically ill neonates [25].

Waldhausen Procedure

In 1966, John A. Waldhausen introduced the Waldhausen procedure (subclavian flap aortoplasty), a technique for addressing coarctation of the aorta and hypoplastic aortic arch or by reconstructing the narrowed aortic segment using the left subclavian artery. This approach minimizes synthetic material use and provides a durable repair in pediatric patients [26].

Congenital diaphragmatic hernia repairs

Bochdalek and Morgagni Hernia Repairs

Congenital diaphragmatic hernias, including Bochdalek and Morgagni hernias, were first described by anatomists Vincent Bochdalek in the 18th century and Giovanni Morgagni in the 19th century, respectively. Surgical repair involves closing the diaphragmatic defect and repositioning herniated organs, with early intervention critical for optimal outcomes [27,28].

Bhende-Pathak Hernia Repair

The Bhende-Pathak hernia, a recently described variant of Bochdalek hernia, exemplifies modern contributions to pediatric surgery. This right-sided diaphragmatic hernia presents with an intrathoracic appendix and concurrent patent ductus arteriosus, highlighting the need for individualized surgical approaches for complex anatomical anomalies [29,30].

Discussion

The evolution of eponymous cardiovascular surgeries reflects the transformative impact of surgical innovation on patient outcomes [31]. Table 1 provides a chronological overview of major eponymous cardiovascular surgeries for thoracic, congenital, and pediatric heart diseases [3,5-7,9,11,13-16,18-21,23-25,27-30].

**Table 1:** Chronological development of eponymous procedures in cardiovascular pediatric surgery CDH, congenital diaphragmatic hernia; CHD, congenital heart disease; PA, pulmonary artery; LPA, left pulmonary artery; TGA, transposition of great arteries; D-TGA, dextro-transposition of the great arteries; SVC, superior  vena cava; RPA, right pulmonary artery; VSD, ventricular septal defect; PS, pulmonary stenosis; HLHS, hypoplastic left heart syndrome; IVC, inferior vena cava; RV, right ventricle; DKS, Damus-Kaye-Stansel procedure; BTT, Blalock-Taussig-Thomas

Procedure	Year	Surgeon(s)	Primary indication	Technical features	Current status
Morgagni hernia repair [28]	1769	Giovanni Morgagni	Anterior diaphragmatic defect	Closure of the anterior defect	Standard approach for anterior hernias
Bochdalek hernia repair [27]	1848	Vincent Alexander Bochdalek	Posterolateral diaphragmatic defect	Repair of the posterior defect	Standard for congenital CDH
Blalock-Taussig shunt [3]	1944	Alfred Blalock, Helen Taussig, Vivien Thomas	Tetralogy of Fallot	Subclavian-to-PA anastomosis	Modified version widely used
Potts shunt [5]	1946	Willis Potts	Cyanotic CHD	Descending aorta-to-LPA anastomosis	Historical interest only
Senning operation [21]	1957	Åke Senning	D-TGA	Atrial switch using native tissue	Limited use in specific cases
Glenn procedure [7]	1958	William Glenn	Single ventricle physiology	SVC-to-PA anastomosis	Modified to bidirectional Glenn
Waterston shunt [6]	1962	David Waterston	Tetralogy of Fallot	Ascending aorta-to-RPA shunt	Rarely used
Mustard procedure [20]	1964	William T. Mustard	D-TGA	Atrial switch with baffle	Historical interest
Rashkind procedure [25]	1966	William Rashkind	D-TGA, mixing	Balloon atrial septostomy	Still used in specific cases
Subclavian flap aortoplasty [26]	1966	Waldhausen JA	Coarctation of the aorta, Hypoplastic aortic arch	Left subclavian artery used to reconstruct the narrowed segment of the aorta	Durable repair in pediatric patients, minimizes synthetic material use
Ross procedure [19]	1967	Donald Ross	Aortic valve disease	Pulmonary autograft	Selected young patients
Bentall procedure [23]	1968	Hugh Bentall, Antony De Bono	Aortic root disease	Composite root replacement	Standard for root pathology
Rastelli procedure [18]	1969	Giancarlo Rastelli	Complex TGA/VSD/PS	Intracardiac tunneling	Active use
Fontan procedure [9]	1971	Francis Fontan	Tricuspid atresia	Total cavopulmonary connection	Standard staged approach
Damus-Kaye-Stansel [15]	1975	Paul S. Damus, Michael Kaye, Horace C. Stansel	Outflow obstruction	PA-to-aorta anastomosis	Utilized as part of stage 1 Norwood surgery, which includes atrial septectomy, DKS, BTT/Sano shunt, and aortic reconstruction.
Jatene procedure [16]	1975	Adib Jatene	D-TGA	Arterial switch operation	Current standard for TGA
Lecompte procedure [24]	1981	Yves Lecompte	TGA variants	PA anterior translocation	Standard modification
Norwood procedure [13]	1983	William Norwood	HLHS	Stage 1 palliation	Standard approach
Kawashima procedure [11]	1984	Yasunaru Kawashima	Single ventricle with interrupted IVC	Bilateral bidirectional Glenn	Selected cases
Sano modification [14]	2003	Shunji Sano	HLHS Norwood modification	RV-PA conduit	Widely adopted variant
Bhende-Pathak repair [29,30]	2024	Vishal V. Bhende, Haryax Pathak	Pediatric variant of congenital Bochdalek Hernia	Repair of the posterior defect	Select cases

These procedures highlight the ingenuity and dedication of pioneering surgeons who addressed some of the most challenging congenital and acquired cardiac conditions. The collaborative efforts of surgeons, cardiologists, and researchers have been instrumental in advancing the field and improving outcomes for patients with complex cardiovascular diseases [32].

Despite their historical significance, many eponymous procedures have been modified or replaced by newer techniques as understanding of pathophysiology and surgical technology has progressed. For example, the Jatene procedure has largely supplanted the Mustard and Senning operations due to its anatomic correction of transposition of TGA, offering superior long-term outcomes [16]. Similarly, refinements in the Fontan and Glenn procedures have improved survival rates and reduced complications in single-ventricle physiology [33].

The emergence of catheter-based interventions and hybrid procedures underscores the dynamic nature of cardiovascular surgery. Techniques such as the Rashkind balloon atrial septostomy paved the way for minimally invasive interventions, demonstrating the potential of integrating interventional cardiology with traditional surgical approaches [25]. Advances in three-dimensional (3D) imaging and surgical planning, including 3D printing for preoperative modeling, continue to enhance the precision and efficacy of surgical interventions. In congenital heart defect repair, 3D-printed models have been used to simulate surgical procedures, improving patient outcomes. These models also serve as valuable tools for patient education and surgical team training, fostering a comprehensive understanding of planned interventions [34,35].

**Figure 1:**
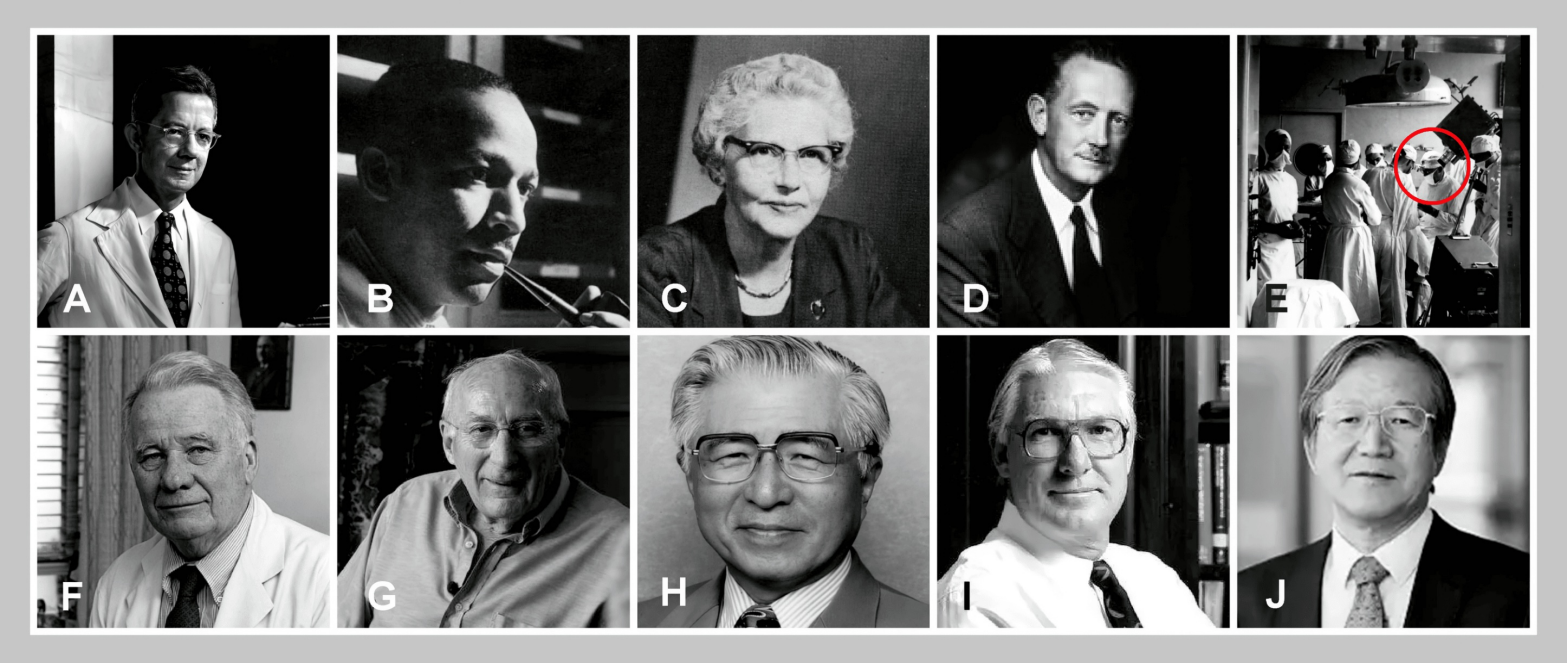
A selection of authors and researchers who have contributed to eponymous cardiovascular and thoracic surgeries. Legend: (A) Alfred Blalock [36] (Image credit: Yousuf Karsh, used with permission). (B) Vivien Thomas [31] (Image credit: Victor C. Baum, Pediatric cardiac surgery: an historical appreciation, Pediatric Anesthesia, volume 16, Issue 12, p. 13, 2006, John Wiley and Sons, reproduced with permission), (C) Helen Taussig [31] (Image credit: Victor C. Baum, Pediatric cardiac surgery: an historical appreciation, Pediatric Anesthesia, volume 16, issue 12, p. 13, 2006, John Wiley and Sons, reproduced with permission), (D) Willis J Potts [37] (Image credit: The image of Dr. Willis Potts is sourced from the US National Library of Medicine, which attributes it to Northwestern University. (E) David Waterston [38] (circled; Image credit: Keystone Press/Alamy Stock Photo, used with permission), (F) William Glenn [39] (Image credit: reprinted from the Lancet, volume 361, Pearce Wright, William WL Glenn, p. 2089, Copyright 2003, with permission from Elsevier), (G) Francis Fontan [40] (Image credit: photo by Edouard Fontan, used under CC BY-SA 4.0), (H) Yasunaru Kawashima [41] (Image credit: Hideki Uemura, Yasunaru Kawashima, Cardiology in the Young, volume 13, issue 1, pp. 84-94, 2005 © Cambridge University Press, reproduced with permission), (I) William Norwood [42] (Image credit: reprinted from Seminars in Thoracic and Cardiovascular Surgery, volume 28, issue 3, Thomas L. Spray, Stephanie Fuller, Christopher E. Mascio, J. William Gaynor, Cardiovascular Surgery at The Children’s Hospital of Philadelphia, pp. 626-633, Copyright 2016, with permission from Elsevier), (J) Shunji Sano [43] (Image credit: Shunji Sano, used with permission).

**Figure 2:**
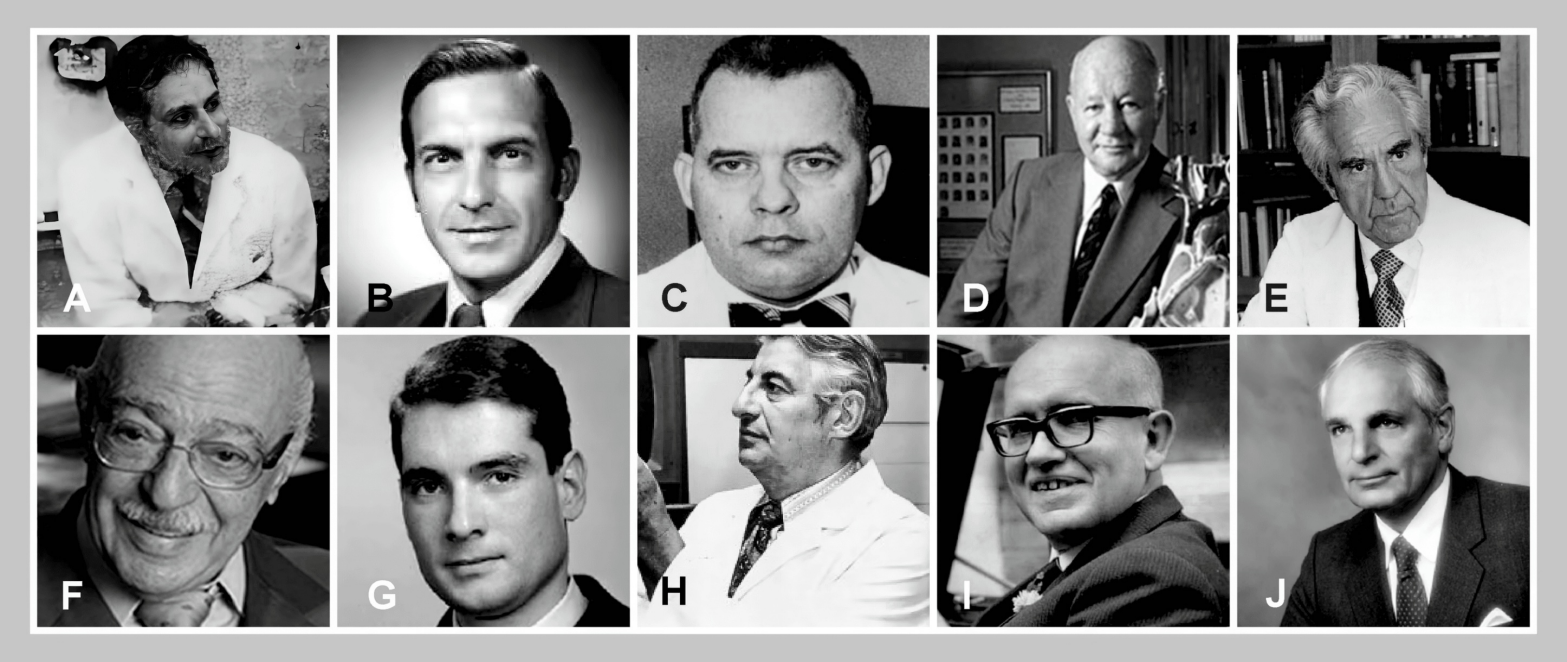
A selection of authors and researchers who have contributed to eponymous cardiovascular and thoracic surgeries (continued). (A) Paul S. Damus [44] (Image credit: © Fred M. Wu, used with permission), (B) Michael Peter Kaye [45] (Image credit: Reprinted from the Journal of Heart and Lung Transplantation, volume 37, issue 4, Stuart Jamieson, Dr. Michael Peter Kaye, p. 534, Copyright 2018, with permission from Elsevier), (C) HC Stansel [46] (Image credit: the image of Dr. Horace Stansel is sourced from the Department of Surgery Images, Yale School of Medicine), (D) William T. Mustard [47] (Image credit: Canadian Medical Hall of Fame, used with permission), (E) Åke Senning [48] (Image credit: Photo by Åke Senning, used under CC BY-SA 4.0), (F) Adib Jatene [49] (Image credit: reproduced from The BMJ, Adib Jatene, Ned Stafford, volume 350, p. 26, 2015, with permission from BMJ Publishing Group Ltd.), (G) Giancarlo Rastelli [50] (Image credit: reprinted from the Annals of Thoracic Surgery, volume 79, issue 5, Igor E. Konstantinov, Felice Rosapepe, Joseph A. Dearani, Vladimir V. Alexi-Meskishvili, Jia Li, A Tribute to Giancarlo Rastelli, pp. 1819-1823, Copyright 2005, with permission from Elsevier), (H) William Rashkind [42] (Image credit: reprinted from Seminars in Thoracic and Cardiovascular Surgery, volume 28, issue 3, Thomas L. Spray, Stephanie Fuller, Christopher E. Mascio, J. William Gaynor, Cardiovascular Surgery at the Children’s Hospital of Philadelphia, pp. 626-633, Copyright 2016, with permission from Elsevier), (I) Donald Ross [51] (Image credit: reprinted from the Lancet, volume 384, Jeremy Laurance, Donald Nixon Ross, p. 576, Copyright 2014, with permission from Elsevier), (J) John A. Waldhausen [52] (Image credit: Reprinted from the Annals of Thoracic Surgery, volume 148, issue 2, Jeffrey S. Heinle, J. William Gaynor, Historical perspectives of the American Association for Thoracic Surgery: John Anton Waldhausen (1929-2012), pp. 381-386, Copyright 2014, with permission from Elsevier).

**Figure 3:**
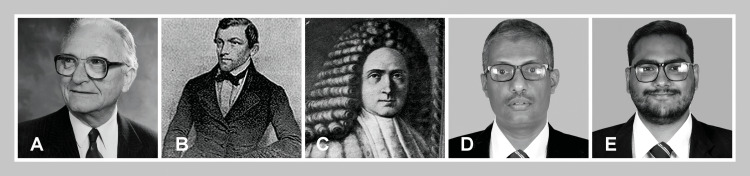
A selection of authors and researchers who have contributed to eponymous cardiovascular and thoracic surgeries (continued). (A) Hugh Bentall [53] (Image credit: Reprinted from The Lancet, Vol. 381, Geoff Watts, Hugh Henry Bentall, p. 720, Copyright 2013, with permission from Elsevier), (B) Vincent Bochdalek [54] (Image credit: Public Domain), (C) Giovanni Morgagni [55] (Image credit: Public Domain), (D) Vishal V. Bhende (Image credit: Image courtesy of the authors, used with permission), and (E) Hаryах Pathak (Image credit: Image courtesy of the authors, used with permission).

**Figure 4:**
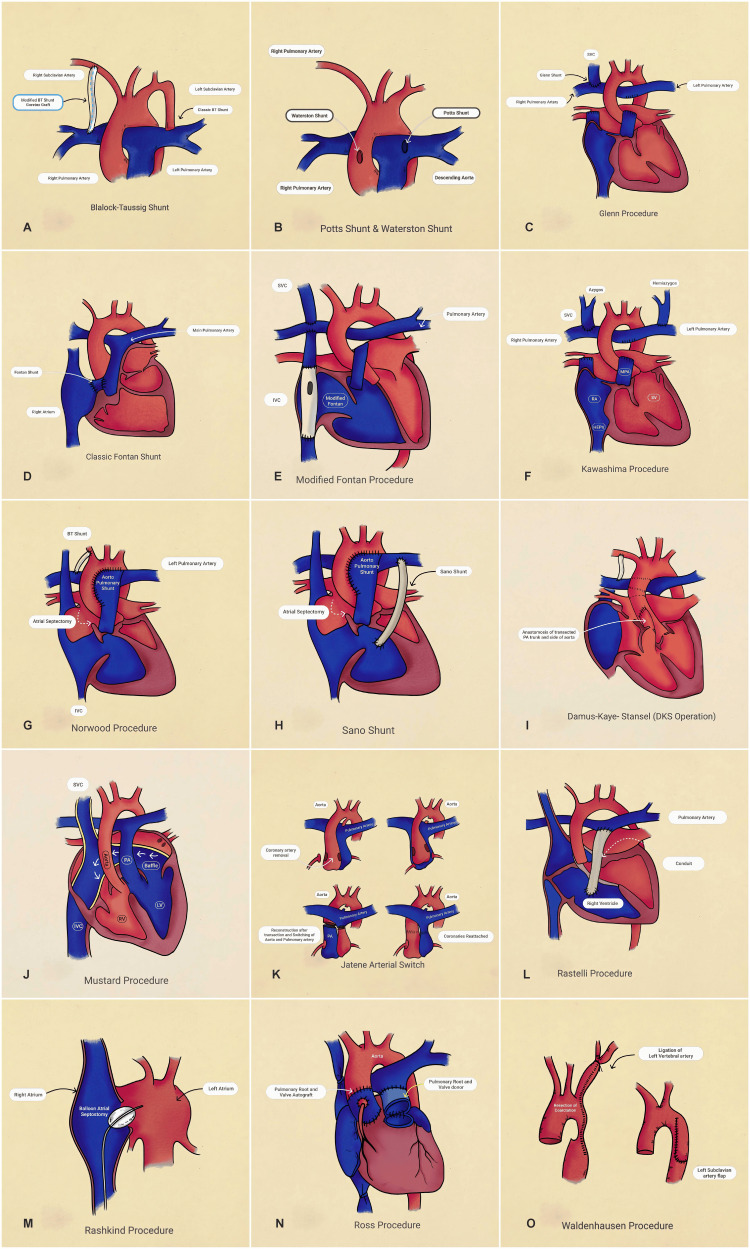
Eponymous cardiovascular surgeries Legend: (A) Blalock-Taussig shunt [3], (B) Pott’s and Waterston shunt [5,6], (C) Glenn procedure [7,8], (D) classic Fontan procedure [9], (E) modified Fontan procedure [10], (F) Kawashima procedure [11,12], (G) Norwood procedure [13], (H) Sano shunt [14], (I) Damus-Kaye-Stansel (DKS) operation [15], (J) Mustard procedure [20], (K) Jatene arterial switch [16], (L) Rastelli procedure [18], (M) Rashkind procedure [25], (N) Ross procedure [19], (O) Waldhausen procedure [26]. Images created by the authors. BT, Blalock-Taussig; SVC, superior vena cava; IVC, inferior vena cava; HEPV, hepatic veins; RA, right atrium; MPA, main pulmonary artery; SV, single ventricle; PA, pulmonary artery; RV, right ventricle; LV, left ventricle; DKS, Damus-Kaye-Stansel.

Another key theme is the adaptability of eponymous procedures to address a broader range of clinical scenarios. For example, the Kawashima modification of the Fontan procedure demonstrates how tailored approaches optimize outcomes for specific anatomical variations [12]. Similarly, the Bhende-Pathak hernia repair illustrates the importance of customizing surgical strategies for unique patient presentations, reinforcing the relevance of eponymous innovations in contemporary practice (Figures 1, 2, 3, 4) [29,30].

As surgical techniques continue to evolve, the legacy of these eponymous pioneers provides a foundation for future innovation. Over time, the iterative improvements and adaptations of these procedures underscore the importance of research, collaboration, and the willingness to challenge established paradigms. The field of cardiovascular surgery remains a testament to human ingenuity and the relentless pursuit of better patient outcomes.

Limitations

This review provides a comprehensive examination of eponymous procedures in cardiovascular and pediatric surgery; however, it is not exhaustive. Several significant procedures that have shaped the field may not be included due to space constraints or limited widespread clinical use. In addition, many recently developed procedures do not yet bear eponymous names despite their importance in advancing surgical techniques. The selection of procedures discussed in this review was guided by historical relevance, frequency of clinical application, and impact on congenital and pediatric cardiac surgery. As a result, the omission of certain techniques may lead to an incomplete representation of the full spectrum of cardiovascular surgical advancements.

Furthermore, the evolution of surgical techniques has led to modifying or replacing several eponymous procedures. Many early palliative shunts, such as the Potts and Waterston shunts, are no longer commonly performed due to improved definitive repair techniques. Thus, while the eponymous procedures highlighted in this review have played a significant role in the history of cardiovascular surgery, some are no longer widely used in current practice. This reflects the dynamic nature of surgical innovation, where techniques continue to evolve alongside advancements in understanding congenital heart disease and surgical intervention.

## Conclusions

Eponymous cardiovascular surgeries represent critical milestones in the history of medicine, reflecting the ingenuity and perseverance of the surgeons who developed them. From the Blalock-Taussig shunt to the Fontan procedure, these techniques have transformed the management of congenital and pediatric heart diseases, offering hope to countless patients and families. While some eponyms may become less common as newer techniques emerge, their legacy will continue to inspire future clinicians and researchers. By building on the foundations established by these eponymous procedures, pediatric cardiovascular surgery will continue to advance, improving outcomes and quality of life for patients with complex heart diseases.

## References

[1] Reller MD, Strickland MJ, Riehle-Colarusso T, Mahle WT, Correa A (2008). Prevalence of congenital heart defects in metropolitan Atlanta, 1998-2005. J Pediatr.

[2] Hoffman JI, Kaplan S (2002). The incidence of congenital heart disease. J Am Coll Cardiol.

[3] Blalock A, Taussig HB (1984). Landmark article May 19, 1945: the surgical treatment of malformations of the heart in which there is pulmonary stenosis or pulmonary atresia. By Alfred Blalock and Helen B. Taussig. JAMA.

[4] Yuan SM, Shinfeld A, Raanani E (2009). The Blalock-Taussig shunt. J Card Surg.

[5] PO WJ, SM S, GI S (1946). Anastomosis of the aorta to a pulmonary artery; certain types in congenital heart disease. J Am Med Assoc.

[6] WA DJ (1962). Treatment of Fallot's tetralogy in children under 1 year of age [Article in Czech]. Rozhl Chir.

[7] GL WW (1958). Circulatory bypass of the right side of the heart. IV. Shunt between superior vena cava and distal right pulmonary artery; report of clinical application. N Engl J Med.

[8] Allgood NL, Alejos J, Drinkwater DC (1994). Effectiveness of the bidirectional Glenn shunt procedure for volume unloading in the single ventricle patient. Am J Cardiol.

[9] Fontan F, Baudet E (1971). Surgical repair of tricuspid atresia. Thorax.

[10] Chin AJ, Whitehead KK, Watrous RL (2010). Insights after 40 years of the fontan operation. World J Pediatr Congenit Heart Surg.

[11] Kawashima Y, Kitamura S, Matsuda H, Shimazaki Y, Nakano S, Hirose H (1984). Total cavopulmonary shunt operation in complex cardiac anomalies. A new operation. J Thorac Cardiovasc Surg.

[12] Setyapranata S, Brizard CP, Konstantinov IE, Iyengar A, Cheung M, d'Udekem Y (2011). Should we always plan a Fontan completion after a Kawashima procedure?. Eur J Cardiothorac Surg.

[13] Norwood WI, Lang P, Hansen DD (1983). Physiologic repair of aortic atresia-hypoplastic left heart syndrome. N Engl J Med.

[14] Sano S, Ishino K, Kawada M (2003). Right ventricle-pulmonary artery shunt in first-stage palliation of hypoplastic left heart syndrome. J Thorac Cardiovasc Surg.

[15] McElhinney DB, Reddy VM, Silverman NH (1997). Modified Damus-Kaye-Stansel procedure for single ventricle, subaortic stenosis, and arch obstruction in neonates and infants: midterm results and techniques for avoiding circulatory arrest. J Thorac Cardiovasc Surg.

[16] Jatene AD, Fontes VF, Paulista PP, Souza LC, Neger F, Galantier M, Sousa JE (1976). Anatomic correction of transposition of the great vessels. J Thorac Cardiovasc Surg.

[17] Nakamura M, Nishioka M (2023). Arterial switch operation for complete levotransposition of the great arteries. Ann Thorac Surg Short Rep.

[18] Rastelli GC, Wallace RB, Ongley PA (1969). Complete repair of transposition of the great arteries with pulmonary stenosis. A review and report of a case corrected by using a new surgical technique. Circulation.

[19] Ross D (1967). Replacement of aortic and mitral valves with a pulmonary autograft. Lancet.

[20] MU WT (1964). Successful two-stage correction of transposition of the great vessels. Surgery.

[21] SE A (1959). Surgical correction of transposition of the great vessels. Surgery.

[22] Barron DJ, Jones TJ, Brawn WJ (2011). The Senning procedure as part of the double-switch operations for congenitally corrected transposition of the great arteries. Semin Thorac Cardiovasc Surg Pediatr Card Surg Annu.

[23] Bentall H, De Bono A (1968). A technique for complete replacement of the ascending aorta. Thorax.

[24] Lecompte Y, Neveux JY, Leca F, Zannini L, Tu TV, Duboys Y, Jarreau MM (1982). Reconstruction of the pulmonary outflow tract without prosthetic conduit. J Thorac Cardiovasc Surg.

[25] Rashkind WJ, Miller WW (1966). Creation of an atrial septal defect without thoracotomy. A palliative approach to complete transposition of the great arteries. JAMA.

[26] Waldhausen JA, Nahrwold DL (1966). Repair of coarctation of the aorta with a subclavian flap. J Thorac Cardiovasc Surg.

[27] Shin MS, Mulligan SA, Baxley WA, Ho KJ (1987). Bochdalek hernia of diaphragm in the adult. Diagnosis by computed tomography. Chest.

[28] Comer TP, Clagett OT (1966). Surgical treatment of hernia of the foramen of Morgagni. J Thorac Cardiovasc Surg.

[29] Bhende VV, Bhatt MH, Patel VB, Tandon R, Krishnakumar M (2024). A tale of two congenital lesions: a case report of congenital diaphragmatic hernia and congenital heart disease managed by successful surgical outcome with review of the literature (Bhende-Pathak hernia). Cureus.

[30] Das D (2024). Congenital cardiac surgery : innovations from India. Ann Pediatr Cardiol.

[31] Baum VC (2006). Pediatric cardiac surgery: an historical appreciation. Paediatr Anaesth.

[32] Buethe J, Ashwath RC, Rajiah P (2015). Eponymous cardiovascular surgeries for congenital heart diseases--imaging review and historical perspectives. Curr Probl Diagn Radiol.

[33] Grandjean JG (2007). Hybrid cardiac procedure: the ultimate cooperation. Neth Heart J.

[34] Segaran N, Saini G, Mayer JL, Naidu S, Patel I, Alzubaidi S, Oklu R (2021). Application of 3D printing in preoperative planning. J Clin Med.

[35] Henn MC, Mokadam NA (2021). Three-dimensional printing to plan intracardiac operations. JTCVS Tech.

[36] (2025). Dr. Alfred Blalock. https://karsh.org/photographs/dr-alfred-blalock-3/.

[37] Images from the History of Medicine (IHM) (2025). Images from the History of Medicine (IHM): Willis J. Potts. Northwestern University. Medical School. Chicago. 1955.

[38] London operation performed Mr David (2025). London operation performed Mr. David Waterston TV camera Stock Photos and Images. https://www.alamy.com/stock-photo/london-operation-performed-mr-david-waterston-tv-camera.html?sortBy=relevant.

[39] Wright Pearce (2003). William W L Glenn obituary. Lancet.

[40] (2025). Wikipedia: Francis Fontan. https://en.wikipedia.org/w/index.php?title=Francis_Fontan&oldid=1279876885.

[41] Uemura H (2003). Yasunaru Kawashima. Cardiol Young.

[42] Spray TL, Fuller S, Mascio CE, Gaynor JW (2016). Cardiovascular surgery at the Children's Hospital of Philadelphia. Semin Thorac Cardiovasc Surg.

[43] Speaker Profile Shunji Sano (2025). Speaker Profile Shunji Sano, MD, PhD. https://www.emedevents.com/speaker-profile/shunji-sano-2.

[44] (2025). Fred Wu, MD. He submitted a manuscript to the @AnnalsThorSurg in December.

[45] Stuart J (2018). In memoriam: Dr. Michael Peter Kaye. J Heart Lung Transplant.

[46] (2025). EliScholar: Dr. Horace Stansel. https://elischolar.library.yale.edu/surgeryimages/12/.

[47] William T (2025). William T. Mustard, MD. https://www.cdnmedhall.ca/laureates/williammustard.

[48] (2025). Wikipedia: Åke Senning. https://en.wikipedia.org/w/index.php?title=%C3%85ke_Senning&oldid=1237422098.

[49] Stafford N (2015). Obituaries: Adib Jatene. BMJ.

[50] Konstantinov IE, Rosapepe F, Dearani JA, Alexi-Meskishvili VV, Li J (2005). A tribute to Giancarlo Rastelli. Ann Thorac Surg.

[51] Laurance J (2014). Obituary: Donald Nixon Ross. Lancet.

[52] Heinle JS, Gaynor JW (2014). Historical perspectives of The American Association for Thoracic Surgery: John Anton Waldhausen (1929-2012). J Thorac Cardiovasc Surg.

[53] Watts G (2013). Obituary: Hugh Henry Bentall. Lancet.

[54] (2025). Vincent Bochdalek. https://en.wikipedia.org/wiki/Vincent_Bochdalek.

[55] Giovanni Battista Morgagni (2025). Giovanni Battista Morgagni. https://en.wikipedia.org/w/index.php?title=Giovanni_Battista_Morgagni&oldid=1271167227.

